# GUILD: GUidance for Information about Linking Data sets†

**DOI:** 10.1093/pubmed/fdx037

**Published:** 2017-03-28

**Authors:** Ruth Gilbert, Rosemary Lafferty, Gareth Hagger-Johnson, Katie Harron, Li-Chun Zhang, Peter Smith, Chris Dibben, Harvey Goldstein

**Affiliations:** 1 Administrative Data Research Centre for England, University College London Great Ormond Street Institute of Child Health, London, UK; 2 Department of Health Services Research and Policy, London School of Hygiene and Tropical Medicine, London, UK; 3 Department of Social Statistics and Demography, University of Southampton, Southampton, UK; 4 Administrative Data Research Centre for Scotland, University of Edinburgh, Edinburgh, UK

**Keywords:** epidemiology, health services, management and policy

## Abstract

Record linkage of administrative and survey data is increasingly used to generate evidence to inform policy and services. Although a powerful and efficient way of generating new information from existing data sets, errors related to data processing before, during and after linkage can bias results. However, researchers and users of linked data rarely have access to information that can be used to assess these biases or take them into account in analyses. As linked administrative data are increasingly used to provide evidence to guide policy and services, linkage error, which disproportionately affects disadvantaged groups, can undermine evidence for public health. We convened a group of researchers and experts from government data providers to develop guidance about the information that needs to be made available about the data linkage process, by data providers, data linkers, analysts and the researchers who write reports. The guidance goes beyond recommendations for information to be included in research reports. Our aim is to raise awareness of information that may be required at each step of the linkage pathway to improve the transparency, reproducibility, and accuracy of linkage processes, and the validity of analyses and interpretation of results.

## Introduction

Data linkage is increasingly used to bring together electronic records containing information from different sources about an individual, organization or location. Linkage offers  a relatively quick and low cost means of capturing information from large administrative data sets for service planning, delivery and evaluation, surveys and censuses, and research. Data linkage centres have been established in many countries, building on early exemplars of linking administrative data for population-based research in the Nordic countries, Manitoba, Western Australia and Scotland (http://www.ipdln.org/data-linkage-centres). For example, the UK government has invested in national networks for health informatics research (http://www.farrinstitute.org/) and in social research using administrative data (https://adrn.ac.uk/).

**Fig. 1:**
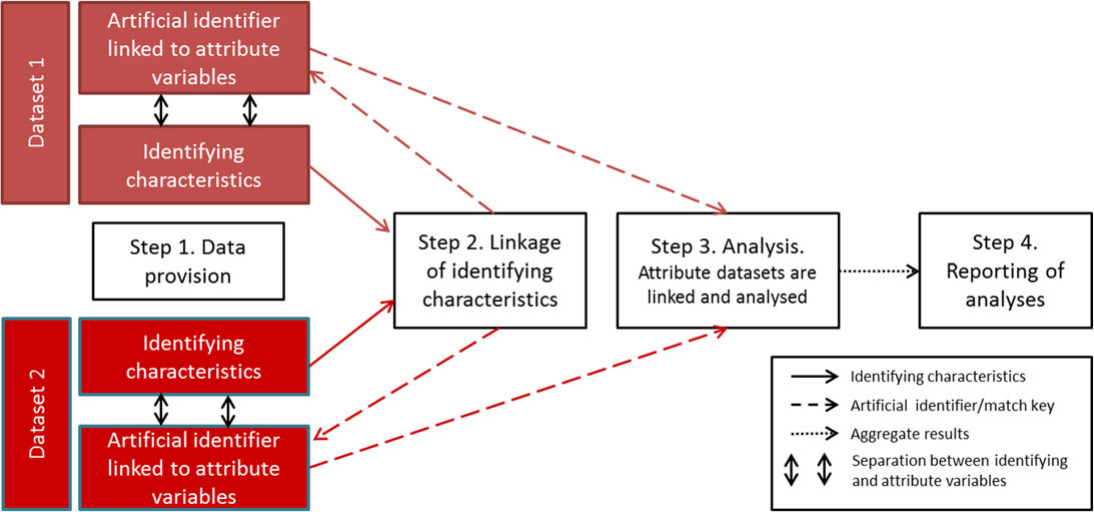
Steps in the data linkage pathway.

Research using linked data is fast becoming a powerful source of evidence to drive policy, practice and biomedical and social sciences.^1^ For example, the USA recently passed legislation to mandate sharing of administrative and survey data with the US Census Bureau for research for evidence-based policy.^2,3^ However, there is growing evidence that important elements of data processing before, during and after linkage, can introduce error and lead to biased results.^1,4,5^ The recent RECORD statement and an earlier framework for reporting recommend information relevant to linkage that should be included in reports of research based on routinely collected health data.^1,6,7^ In practice, however, such information is rarely available to researchers. Lack of information is partly because different processes along the data linkage pathway are performed by different agencies (Fig. 1). Such fragmentation creates barriers to sharing of information about data processing, prevents analyses that take linkage error into account and can limit understanding of the impact of data quality and linkage error on the results of analyses.

The GUILD guidance addresses this lack of understanding by recommending information that could be made available at each step of the data linkage pathway, by data providers, data linkers, analysts and those writing reports. GUILD guidance does not set minimum standards or criteria for information that should be provided nor is it a checklist or protocol. The aim is to set out principles, to raise awareness, and empower data linkers, analysts, researchers and users of evidence to request and use information to assess linkage error and its impact on results. Linkage error is just one of the consequences of poor data quality or missing data. Analysts have a range of methods for dealing with data quality issues, including linkage error, provided they are made aware of the problem.

### Linkage error

Errors in linkage typically occur where there is no unique identifier across different data sets. In the UK, for example, education, health and tax records use different personal identifiers: a pupil ID, National Health Service (NHS) number and National Insurance (NI) number, respectively. Linkage between these data sources, therefore, relies on other common or quasi-identifying characteristics such as name, sex, date of birth and postcode. There is considerable potential for linkage error as some individuals share the same identifying characteristics, identifiers may be entered incorrectly, or different identifiers may be used across data sets (and over time) for the same person. Linkage error occurs in two ways: false-matches are made where two records are linked but do not belong to the same individual, and missed-matches occur when two records that do belong to the same individual fail to link (see Supplementary data, Appendices 1 and 2).^8^ Even small amounts of false- or missed-matches can produce substantially biased results, particularly in data belonging to specific sub-groups of the population, for example, young people, ethnic minorities or the homeless.^9–14^

Fragmentation of data processing can make it hard for data linkers and analysts to have the information needed to assess or take into account the impact of linkage error on results. It is common practice for data linkers to keep identifiers (e.g. NHS number or date of birth), separate from attributes (such as information on health, finance or education). This ‘separation principle’ is used to avoid disclosure during the linkage process (Fig. 1). The identifying characteristics are used only for linkage, which may be done by a separate agency (or third party). The attribute data are linked for analysis using an artificial identifier that cannot be used to identify individuals in the real-world (Fig. 1).

While the separation principle might reduce the risk of identification, it can increase the risk of biased analyses.^14^ Linkers and analysts may be unaware of important groups who are disproportionately affected by linkage error if information is not shared between them. For example, when linking mother and baby data to study infant mortality, babies who die in the first day or two of life may be less likely to be linked because their name or NHS number had not been allocated before death.^15,16^ Data linkers will be unaware of this problem as death is an attribute that is not included with the identifiers used for linkage. Unless information on linkage error is shared with the analyst and incorporated into results, mortality rates could be underestimated. Another example is the calculation of readmission rates for monitoring performance of hospitals. Incorrect or missing patient identifiers are likely to lead to underestimated readmission rates: hospitals with poor quality identifiers will appear to perform better. Provided information on data quality indicators associated with missed-matches or false-matches is made available, linkage error can be mitigated by adaptations to the linkage method, analyses or both.^13,14^ The GUILD guidance highlights elements of the linkage pathway when error can be introduced and recommends information that can be used to assess or account for linkage error without breaching privacy.

### Guidance development

The GUILD guidance was developed by a core group of UK data linkage experts. In March 2015, we held a meeting with eight experts from the Office for National Statistics and from four academic institutions, chosen for their expertise and experience in data linkage across multiple disciplines including social statistics, health care, demography and education. A core group of four experts reviewed previous guidance, reviews of linkage accuracy studies, and other studies reporting sources of bias along the data linkage pathway,^1,4,5,7^ and drafted initial statements, which were revised following discussion at three face-to-face meetings with the UK expert group. The group debated the steps in the linkage pathway that can increase or mitigate linkage error and its impact on results. No formal process was used to achieve consensus. The main item of contention related to the acceptability of statistical disclosure controls that degrade the quality and utility of the data prior to analysis (Supplementary glossary, Appendix S1).^17,18^

Drafts of the recommendations were reviewed by a wider team of UK linkage experts in June 2016 (24 UK experts). We also presented the guidance at an international workshop on data linkage in September 2016 and subsequently held a face-to-face meeting of six international and three UK experts to discuss revisions to the guidance (all contributing experts are listed in the acknowledgements).^19^

In the next section and in Table 1, we propose items of information prioritized by the linkage experts for sharing at each step of the linkage pathway (Fig. 1). Such information could be included in reports of analyses using linked data, or as Supplementary data (e.g. online Appendices).^20^

**Table 1:** GUILD guidance information to be shared before, during and after data linkage

Item	Concept	Guidance
Step 1	Data provision	
1a	Population included in the data set	Data providers should give details of the population included in the data set (e.g. everyone registered with a GP), the geographic coverage of the data (e.g. England and Wales), the number of records in each source data set and how any ‘opt-outs’ were dealt with
1b	Linkability of the data set	Details should be shared about how the data were generated (e.g. face-to-face), processed (e.g. a self-entered form or entered by an administrator) and quality controlled (e.g. manually checked), including how identifying characteristics were
1b(i)		– Collected and allocated
1b(ii)		– Updated as further personal data were collected, and dates of most recent updates
1b(iii)		– Checked and cleaned, including any validation rules
1b(iv)		– Replaced with artificial identifiers to reduce disclosure before being released for linkage
Step 2	Data linkage	
2a	Descriptions of linkage processes	Data linkers should provide descriptions of how the linkage was done including:
2a(i)		– A clear description of the data sources and identifying characteristics used for linkage, details of how identifiers were cleaned and validated before linkage, patterns of missingness, the expected range of values after cleaning, and how any de-duplication was performed.
2a(ii)		– Details of any transformation or replacement with artificial identifiers before linkage
2a(iii)		– A detailed description of the method (or algorithm) used for linkage, whether it was rule-based (e.g. deterministic) or score-based (e.g. probabilistic linkage), and how multiple linkages were handled
2a(iv)		– A detailed description of any new derived variables that were introduced during the linkage process (e.g. confidence level or probability of linkage or link score)
2a(v)		– Details of any blocking or grouping methods used for score-based linkage and how match scores were derived
2b	Record-level indicators of the linkage process	Data linkers should provide analysts with record-level indicators of the data linkage process to enable adjustments for linkage error in the analyses. Indicators could include the pass-ID (the step in a rule-based linkage process when a pair of records linked), or match scores (e.g. match weights used in probabilistic linkage)
2c	Aggregate linkage results	Data linkers should make available descriptions, tables and flow diagrams depicting linkage accuracy for each linkage undertaken. These should include:
2c(i)		– A description of the number of records that were linked and unlinked in each of the source files
2c(ii)		– A table comparing the aggregate characteristics of individuals in the linked and unlinked records for each source data set (defined by the analyst in agreement with the data linker)
2c(iii)		– A description of the ‘representativeness’ of the linked data set to each source data set, for example, including weights that can be applied to allow grossing up the linked data set to better represent the source data sets
2c(iv)		– A flow diagram to represent the steps in linkage and numbers involved at each step
2d	Generic reports of linkage accuracy	The data linker should report generic information about the quality of linkage carried out. This should include:
2d(i)		– Estimates of linkage error rates based on regular quality monitoring of linkage accuracy. For example, measures of the sensitivity and specificity for the algorithm used
2d(ii)		– Details of how error rates were estimated, for example, by comparing linked records with a reference data set
2e	Descriptions of disclosure controls	Data linkers should describe any statistical disclosure controls used to reduce identifiability of linked data prior to release to data analysts
2f	Overview of data linkage	Data linkers should establish systems to improve the quality of linkage studies, for example, by publishing a database detailing the data linkages undertaken with links to publications. The advisory and approvals structure for data linkage should include experts who can scrutinize the impact of linkage processes on results of analyses
Step 3	Data analyses	Data analysts should assess and report on the quality of the linked data used for analyses
3a	Account for linkage error	Analysts should report how analyses took into account linkage error, including:
3a(i)		– How record-level indicators of the linkage process or aggregate measures reflecting linkage quality were used for adjustments, including underlying assumptions and methods used
3a(ii)		– Uncertainty analyses of the effects of linkage errors
3a(iii)		– Sensitivity analyses to determine the impact of assumptions used in the analyses
Step 4	Reporting study findings	Reports of linkage studies should, where possible, include items in Steps 1–3, building on the RECORD statement for research reports (Supplementary data, Appendix 3)^6^

### Step 1. Data provision—the generation, processing and quality control of the source data for linkage

The data provider should publish or otherwise share information to explain how the data set was created and maintained (Table 1, Step 1a, 1b(i–iv)). In some cases, data providers may need to obtain this information from the service that generated the data. The way data are collected, cleaned and standardized can influence the accuracy of the data and any subsequent linkage.^21^ Data providers should share information about how unique identifiers (e.g. NHS number, NI Number and driving license number) were generated and validated. Transcription errors, misspellings and missing data in particular can cause false- and missed-matches.^13,22,23^ Information about data cleaning rules and the extent of missing data or errors in identifiers can help identify common scenarios that cause linkage error.^13^ Information should also be provided about any preprocessing of source data sets involving internal linkage of multiple records to the same entity or to remove duplicate records (Table 1, Step 1, 1b(iii)). For example, in Hospital Episodes Statistics (HES) for NHS hospital contacts in England, an algorithm links repeated contacts over time for the same patient.^13,24^ False-matches and missed-matches occurring during this internal linkage can compound subsequent linkage errors when the HES is linked externally to another data set, such as primary care records.^25^ Provided information is shared about internal linkage errors within one or more of the source data sets, data linkers may be able to develop linkage algorithms that minimize the problem.^14^ In addition, information on the rates of false- and missed-matches can be used to adjust results of analyses or to undertake sensitivity analyses.^5^

Data providers or data linkers can replace real-world identifiers with artificial identifiers, i.e. numbers or codes that cannot be traced to the individual or unit (Table 1, Step 1, 1b(iv) or Step 2, 2a(ii)). The aim is to reduce the risk of identification during linkage. A variety of methods can be used, referred to as privacy preserving techniques.^26,27^ For example, the UK Office of National Statistics replaces real-world names and numbers with an artificial identifier after cleaning and standardization of data received from data providers but prior to linkage (Table 1, Step 2, 2a(ii)). This process is irreversible as the artificial identifier cannot be decoded to regenerate the real-world identifiers.^4,28^ Replacement with artificial identifiers prior to linkage is controversial because it makes it difficult to quantify or take into account linkage errors related to certain characteristics, such as names, postcodes or dates.^29^

### Step 2. Data linkage—bringing together records belonging to the same individual, place or organization

The first part of the guidance about data linkage (Table 1, Step 2, 2a–b) relates to the information that should be shared when undertaking linkage of two or more data sets for a specific study or analysis. Data linkers should describe and justify the identifying characteristics (e.g. name, postcode, sex and ethnicity) used in the linkage algorithm. In addition to the data cleaning and validation undertaken by data providers (Table 1, Step 1b, 2ai), data linkers may undertake further cleaning and validation of identifying characteristics used for linkage (Table 1, Step 2, 2ai). Cleaning the data by removing spaces in postcodes or editing dates by imputing information where there are inconsistencies, makes it more likely that two identifying characteristics will agree. Care must be taken, whilst data cleaning could enable data linkage to capture more true matches, it could also make it more likely that two records will falsely link.^25^ The rules used to standardize data should, therefore, be reported in detail, because they influence linkage error.^13^ It is also important to report the proportion of missing data before and after cleaning, and the number of records excluded or changed, for example, because of duplicate records, improbable characteristics (e.g. date of death before birthdate) or not meeting study criteria (Table 1, Step 2, 2a(i and ii)).

Information about methods used to link data should be shared with analysts and where feasible, this information should be published, including details of the linkage algorithm (Table 1, Step 2, 2a(iii)). A common method for data linkage is to first use rule-based matching (e.g. deterministic or exact matching) followed by score-based matching (e.g. probabilistic linkage) to link any remaining records.^30^ Despite evidence that probabilistic linkage produces less biased results than deterministic linkage alone,^31,32^ probabilistic linkage is rarely used for linking administrative data in the UK. However, data linkers in Wales (SAIL), Scotland (eDRIS), Australia, the US and Canada, demonstrate that probabilistic linkage is feasible at scale.^23,33,34^

Data linkers using score-based methods should report how they grouped records that could potentially link—referred to as blocking. (Table 1, Step 2, 2a(iv)). Blocking means that only those records with some degree of similarity are compared, e.g. only those where date of birth agrees.^4^ Blocking aims to reduce processing time, but can cause missed-matches.

The data linker should share record-level information that enables the analyst to take linkage uncertainty into account in analyses (Table 1 Step 2, 2b). This can be done by attaching indicators of match certainty to each comparison pair of matched records. In rule-based linkage, indicators might reflect the step in the algorithm at which the records were linked (e.g. pass-identifier). In score-based linkage, record-level indicators include match-scores (e.g. match weights, probabilities or ranks). The group or block indicator adds information on how uncertainty varies across groups. When score-based linkage is used, information on the optimum threshold for designating links as matches should be shared, and, where possible, a matrix that shows all possible links for each record above the threshold. These record-level indicators can be used to adjust linked data sets, for example by including or excluding links based on the uncertainty of the match as defined by the match-score.^5,35^

Following the production of a linked data set, the data linker should provide a description of linkage accuracy at the aggregate level (Table 1 Step 2, 2c(i–iv)). This could include a comparison of aggregate counts of age, sex and other attributes, and reports of the uniqueness and independence of identifying characteristics used for linkage.^36,37^

Data linkers should provide generic information reflecting regular quality assessments of their linkage processes (Table 1 Step 2, 2d–f), where these are large-scale, ongoing linkages (e.g. all hospitalizations and deaths nationally). In this situation, regular comparisons of samples of linked data to a reference data set where true- and false-matches are known, may be sufficient provided information is reported for important subsections of the population (e.g. infants, elderly) for whom linkage accuracy may vary.^14^ Measures include precision or positive predictive value (a measure of false-matches), sensitivity/recall (a measure of missed-matches) and the *F*-measure (Supplementary data, Appendix S2).^4^

Data linkers should publish their methods for disclosure control of linked data before transmission of linked data to the analyst. For example, data linkers sometimes require grouping of detailed values into broader groupings (e.g. changing exact ages to age bands), suppression of outlying values, or addition of random noise to minimize disclosure risks (Table 1, Step 2, 2e).^17,18,38^ Making information about the linkage processes publicly available can help to develop rigorous methods throughout the data linkage pathway. Data linkers can support transparency, quality and reproducibility of studies and encourage collective learning about linkage error by publishing details of linkages undertaken with links to subsequent study reports (Table 1, Step 2, 2f).

### Step 3. Analyses of the linked data—taking account of linkage error

So far, the guidance has focused on providing the data analyst with the information they need to conduct analyses that take into account sources of error before, during and after linkage (Table 1, Steps 1–3). The analyst should report any evaluation of linkage accuracy against a reference standard and how they used this information in their analyses in meta-data or research reports (see Supplementary data, Appendix 3).

The analyst should report use of record-level indicators of linkage uncertainty (e.g. match weights) in the analyses, for example, whether varying the match score changed the results of analyses (Table 1, Step 3, 3a(ii–iii)).^5,14,35^ An alternative approach is to use match weights for all possible links to select the correct value for the variable of interest (known as prior informed imputation).^4,39^ This method avoids errors that could be incurred by accepting the wrong record as a link. If the analyst does not have record-level indicators of the linkage process, they can adjust for linkage error based on comparisons of the linked data with the unlinked source populations or through external comparisons with expected rates (Table 1, Step 3, 3a(i)).

### Step 4. Reporting the results of analyses of linked data

Reports of studies using linked data should, where possible, include information on items in Steps 1–3. Information should be prioritized to enable users of studies (e.g. journal editors, researchers, policy makers, data providers and linkers and the public) to understand the extent of linkage error and the potential impact on results and reproducibility of analyses.^2,40^ Research reports should continue to use the STROBE guidance, supplemented by the 13-item RECORD statement for specific items of information for observational studies using administrative data, including the four items about data linkage (Supplementary data, Appendix 3).^6^ When publishing results, statistical disclosure controls may prevent publication of potentially disclosive information, such as minimum–maximum ranges and small cell sizes, which could provide insights into linkage error. In these circumstances, potentially disclosive results may need to be restricted to approved users.^41^

## Discussion

### Main findings of this study

GUILD aims to improve the quality of data processing, linkage, analyses and research reports by raising awareness about detailed information that could be shared at each step of the linkage pathway. The guidance also aims to highlight the responsibilities of data providers, linkers and analysts, not just report writers, to make this information available.

### What is already known?

Linkage error can contribute to selection bias or information bias or both, depending on the study design and the way in which linkage is used to generate the variables used in analyses. The STROBE and RECORD reporting guidelines make recommendations about information that should be included in research reports of observational studies based on electronic health data sets but do not provide guidance on potential sources of linkage error.^6,42^

### What this study adds

GUILD highlights the choices and decisions made during data processing that affect linkage error and hence the results of analyses. Sharing information along the data linkage pathway could improve the transparency and reproducibility of research, promote the use of improved methods to address linkage error, and improve the interpretation of studies based on linked data.

### Limitations of the study

Development of the GUILD guidance involved iterative discussions with UK and international linkage experts but did not use formal consensus methods. The scope of GUILD is broad, involving different processes and a variety of agencies, analysts and methods. Further methodological research can inform updates to this guidance and help to prioritize key items of information that should be made available. There is also a need to develop appropriate formats (e.g. meta-data and data sharing agreements) for sharing information about sources of linkage error while preserving the privacy of data entities or individuals.

Linked administrative data are a powerful resource, which is increasingly used to underpin policy, organization of services and research. Transparency throughout the linkage pathway is important to ensure that the validity of this resource is fit-for-purpose.

## Supplementary data

Supplementary data are available at *Journal of Public Health* online.

## Supplementary Material

Supplementary Data
