# Supplementary material for: GUILD: GUidance for Information about Linking Data sets
Source: J Public Health (Oxf). 2017 Mar 28;40(1):191–8. doi: 10.1093/pubmed/fdx037 (PMC5896589; doi:10.1093/pubmed/fdx037)
Supplement: Supplementary Data [file guildguidancesupplementarymaterialfinalv1.docx]

**GUILD Guidance**

**Appendix 1. Glossary**

| **Glossary** | **Description** |
| --- | --- |
| Administrative data. | Data that has been collected (e.g. by a government department) to enable the provision, monitoring and evaluation of services. |
| Algorithm. | A sequence of steps or rules to follow in order to process data or perform calculations, normally used by computers. |
| Anonymization. | Anonymization is the process by which the relationship between an individual and the data about them is broken, so that the individual cannot be identified.(1) Alternative terms include de-identification or pseudo-anonymizations. |
| Artificial identifier | Replacement of real-world identifiers that could be traced to an individual (e.g. NHS number or passport number) with a unique number or code that cannot be used to an individual (or other entity). |
| Attribute data. | The characteristics of interest about the entity, such as earnings or healthcare. Attribute data are recorded as well-defined variables (e.g. column in a database). Attribute data that are non-identifying and not informative for linkage are kept separate from identifying characteristics under the separation principle. |
| Blocking. | A method for reducing the number of data comparisons that need to be made. Records are compared only if they already have a degree of similarity defined by the data linker (e.g. blocking by hospital or date of birth). Only records that belong to the same block can possibly be linked. |
| Block identifier or Blocking key value. | A combination of numbers or letters that identifies the block that each record belongs to. |
| Blocking key. | Defines how blocks are to be formed (e.g. first two letters of surname connected with year of birth).(2) |
| Data error. | A broad term referring to misspelt or incorrectly recorded identifying characteristics, false information or missing information. |
| Data linkage. | The process of linking *records* from two or more databases that refer to the same *entity*. These pairs or groups of records are known as *matches* and can relate to a person, place, business and/or organization.(2) The process of comparing records *records* from two or more databases with the objective to identify pairs or groups of records that refer to the same *entity* is known as data matching. |
| Deterministic linkage. | Two records are designated as matches based on their attributes being the same (e.g. exact match on sex, date of birth and postcode), or highly similar (e.g. match on partial date of birth, exact match on sex and postcode). These matches are determined by a set of rules (an algorithm) created by the data linker. |
| False match. | A record pair that is classified as a match where, however, the two records in the pair refer to two different entities.(2) |
| Identifying characteristics. | Quasi-identifiable variables that directly identify an individual (e.g. name) or that can indirectly be used in combination with others to uniquely identify an individual (e.g. date of birth, sex and postcode). |
| Linkage error. | A generic term referring both to false and missed matches. |
| Linked data. | The product of record linkage, data that has been produced by the record linkage of two or more datasets. |
| M and U probabilities. | Numerical values that represents the probability that two records agree on a variable given they are a true match (m value) and the probability that two records agree on a variable given they are true non-matches (u value).(3) |
| Match scores. | A numerical value that represents the likelihood of two records being a match.(3) |
| Match rates. | The number of linked records out of the total eligible for linkage in one of the source files. |
| Match weights. | A numerical value that is assigned to a certain attribute where the attribute values are the same or similar to each other.(3) This is also known as an agreement weight. Match weights are calculated as the likelihood that two attribute values are in agreement assuming that both records in a candidate record pair correspond to the same entity, divided by the likelihood that two attribute values are in agreement assuming that the two records in a candidate record pair correspond to different entities. |
| Missed match. | A record pair that is classified as a non-match where, however, both records in the pair correspond to the same entity, otherwise known as a false non-match. |
| Negative predictive value (NPV). | The proportion of record pairs classified by the algorithm as non-links that are true non-matches. |
| Pass-ID. | A combination of numbers or letters that identifies the stage in the linkage method that the match was made. For example, a pass-id could relate to a specific step in a rule-based linkage algorithm. |
| Personal data. | Personal data is defined as data which can be used to identify an individual, including when that data is combined with other information. In some countries, personal data has a specific legal definition. |
| Positive predictive value (PPV). | The proportion of record pairs classified by the algorithm as links that are true matches. This is also known as precision. |
| Precision. | See positive predictive value. |
| Probabilistic record linkage. | Records are matched based on the degree of similarity between the linkage variables, expressed explicitly in terms of the relevant probabilities. This is often known as score-based matching. The approach published by Fellegi and Sunter calculates match weights and non-match weights based on error probabilities and frequency distributions of attribute values in the input databases. Candidate record pairs are classified based on their weight vectors into either matches, non-matches, or potential matches, using a threshold-based and pair-wise classification approach.(3) |
| Pseudonymised. | Data in which identifying fields (e.g. names, dates of births and addresses) have been replaced by one or more artificial identifiers to reduce the risk of identification of individuals.(4) |
| Recall. | See sensitivity. |
| Sensitivity. | The proportion of true matches that are correctly classified as links. This is also known as recall. |
| Specificity. | The proportion of true negative matches that are correctly classified as non-links. |
| Statistical disclosure control (SDC). | Methods to measure and reduce the risk of disclosing information on individual entities (e.g.: individuals, households or organizations).(5) SDC can involve changing record level data before analyses (Figure 1, step 3) or aggregate data before reporting of analyses (Figure 1, step 4). SDC before analyses usually involves removal of unique identifiers (e.g. NHS number) and quasi identifying characteristics (e.g. date of birth, postcode). It can also involve changing attribute data to reduce the risk of unique combinations of characteristics that could be used to identify individuals. In this way, SDC can degrade the quality and utility of the data before analysis. SDC is also applied to aggregate data in reports, for example by modifying aggregate results, such as cell sizes containing fewer than 5 individuals (Figure 1, step 4). |
| True match. | A record pair that is classified as a match, where both records in the pair correspond to the same entity. This is also known as a true positive. |
| True non-match. | A record pair that is classified as a non-match, where the two records in the pair correspond to two different entities. This is also known as a true negative. |
| Trusted third party. | An organization that undertakes record linkage using data provided by other organizations. |

**Appendix 2. Quantitative measures of linkage accuracy (2,5)**

|  |  | **True match status** | |
| --- | --- | --- | --- |
|  |  | **Match**  **(record pair is from the same individual)** | **Non-Match**  **(record pair is from different individuals)** |
| **Status after linkage** | **Link** | A: True positive matches | B: False-matches |
|  | **Non-link** | C: Missed matches | D: True negative matches |

Examples of quantitative measures of linkage accuracy are given below.

1. The positive predictive value (PPV) - the proportion of record pairs classified by the algorithm as links that are true matches. Also known as precision.

PPV = A/(A+B)

1. The negative predictive value (NPV) - the proportion of record pairs classified by the algorithm as non-links that are true non-matches.

NPV = D/(D+C)

1. The specificity – the proportion of true negative matches that are correctly classified as non-links.

Specificity = D/(B+D)

1. The sensitivity – the proportion of true matches that are correctly classified as links. Also known as recall.

Sensitivity = A/(A+C)

1. The F-measure – The harmonic mean between positive predictive value and sensitivity. Often used to compare the overall efficiency of a method.

F-measure = 2*(PPV*sensitivity)/(PPV+sensitivity)

**Appendix 3. Items in the RECORD statement relevant to data linkage (6)**

| **Title and abstract**  RECORD 1.3: If linkage between databases was conducted for the study, this should be clearly stated in the title or abstract. Introduction |
| --- |
| **Methods: Participants**  RECORD 6.3: If the study involved linkage of databases, consider use of a flow diagram or other graphical display to demonstrate the data linkage process, including the number of individuals with linked data at each stage. |
| **Methods: Statistical Methods**  RECORD ITEM 12.2: Authors should provide information on the data cleaning methods used in the study.  RECORD ITEM 12.3: State whether the study included person-level, institutional-level, or other data linkage across two or more databases. Linkage techniques and methods used to evaluate linkage quality should be provided. |
| **Results: Participants**  RECORD ITEM 13.1: Describe in detail the selection of the persons included in the study (i.e., study population selection), including filtering based on data quality, data availability and linkage. The selection of included persons can be described in the text and/or by means of the study flow diagram. |
| **Discussion: Limitations**  Discussion RECORD ITEM 19.1: Discuss the implications of using data that were not created or collected to answer the specific research question(s). Include discussion of misclassification bias, unmeasured confounding, missing data, and changing eligibility over time, as they pertain to the study being reported. |

References

1. Tinabo R, Mtenzi F, O'Shea B, editors. Anonymization vs. pseudonymization: Which one is most useful for both privacy protection and usefulness of e-healthcare data. Internet Technology and Secured Transactions; 2009; London. <http://ieeexplore.ieee.org/xpls/abs_all.jsp?arnumber=5402501> (accessed 21.3.2017)

2. Christen P. Data Matching: Concepts and techniques for record linkage, entity resolution, and duplicate detection. London: Springer-Verlag Berlin Heidelberg; 2012.

3. Fellegi I, Sunter A. A theory for record linkage. Journal of the American Statistical Association. 1969;64(328):1183-210.

4. Hundepool A; Domingo-Ferrer J FL, Giessing S, Schulte Nordholt E, Spicer K, de Wolf PP. . Statistical Disclosure Control. Chichester, UK: Wiley; 2012.

5. Harron K, Goldstein H, Dibben C. Methodological Developments in Data Linkage: John Wiley & Sons; 2015.

6. Benchimol EI, Smeeth L, Guttmann A, Harron K, Moher D, Petersen I, et al. The REporting of studies Conducted using Observational Routinely-collected health Data (RECORD) Statement. PLoS Med. 2015;12(10):e1001885.
